# The pathological risk score: A new deep learning‐based signature for predicting survival in cervical cancer

**DOI:** 10.1002/cam4.4953

**Published:** 2022-06-28

**Authors:** Chi Chen, Yuye Cao, Weili Li, Zhenyu Liu, Ping Liu, Xin Tian, Caixia Sun, Wuliang Wang, Han Gao, Shan Kang, Shaoguang Wang, Jingying Jiang, Chunlin Chen, Jie Tian

**Affiliations:** ^1^ Beijing Advanced Innovation Center for Big Data‐Based Precision Medicine, School of Medicine and Engineering Beihang University Beijing China; ^2^ CAS Key Laboratory of Molecular Imaging, Beijing Key Laboratory of Molecular Imaging, the State Key Laboratory of Management and Control for Complex Systems Institute of Automation, Chinese Academy of Sciences Beijing China; ^3^ Department of Obstetrics and Gynecology Nanfang Hospital, Southern Medical University Guangzhou China; ^4^ School of Artificial Intelligence University of Chinese Academy of Sciences Beijing China; ^5^ Department of Obstetrics and Gynecology The Second Affiliated Hospital of He' nan Medical University Zhengzhou China; ^6^ Department of Gynecology Fourth Hospital Hebei Medical University Shijiazhuang China; ^7^ Department of Gynecology Yantai Yuhuangding Hospital Yantai China; ^8^ Key Laboratory of Big Data‐Based Precision Medicine (Beihang University) Ministry of Industry and Information Technology Beijing China

**Keywords:** cervical cancer, deep learning, disease‐free survival, overall survival, whole slide image

## Abstract

**Purpose:**

To develop and validate a deep learning‐based pathological risk score (RS) with an aim of predicting patients' prognosis to investigate the potential association between the information within the whole slide image (WSI) and cervical cancer prognosis.

**Methods:**

A total of 251 patients with the International Federation of Gynecology and Obstetrics (FIGO) Stage IA1–IIA2 cervical cancer who underwent surgery without any preoperative treatment were enrolled in this study. Both the clinical characteristics and WSI of each patient were collected. To construct a prognosis‐associate RS, high‐dimensional pathological features were extracted using a convolutional neural network with an autoencoder. With the score threshold selected by X‐tile, Kaplan–Meier survival analysis was applied to verify the prediction performance of RS in overall survival (OS) and disease‐free survival (DFS) in both the training and testing datasets, as well as different clinical subgroups.

**Results:**

For the OS and DFS prediction in the testing cohort, RS showed a Harrell's concordance index of higher than 0.700, while the areas under the curve (AUC) achieved up to 0.800 in the same cohort. Furthermore, Kaplan–Meier survival analysis demonstrated that RS was a potential prognostic factor, even in different datasets or subgroups. It could further distinguish the survival differences after clinicopathological risk stratification.

**Conclusion:**

In the present study, we developed an effective signature in cervical cancer for prognosis prediction and patients' stratification in OS and DFS.

## Graphical abstract

This is a deep learning‐based study that encoded the high dimensional features from whole slide image to establish an independent prognostic risk score, which can further distinguish survival differences after clinicopathological risk stratification.

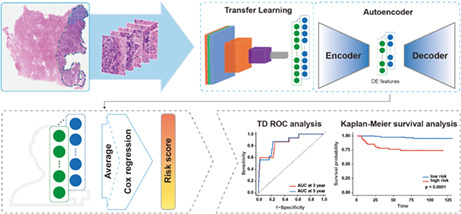

## 1 INTRODUCTION

In 2020, there were 604,127 new cases and 341,831 deaths related to cervical cancer worldwide.^1^ Considering the current guidelines in cancer treatment, most patients with early‐stage cervical cancer can be cured by radical surgery through appropriate adjuvant radiotherapy according to postoperative pathological risk factors.^2^ Although the prognosis is favorable in early cervical cancer patients with standard treatment, there are still numerous patients with nodal metastases and/or locally advanced tumors will progress.^3^ Therefore, if the outcome of the disease has been identified at an early stage, individualized follow‐up and treatment plans will help to improve the prognosis of patients with cervical cancer.^4^

Pathological hematoxylin–eosin (H&E) staining was the gold standard for cervical cancer diagnosis. The method of quantitatively describing the microenvironment of cervical cancer has been highly recognized in exploring novel prognostic signatures, since it could describe the pathological characterization of the tumor. Some recent studies calculated the tumor‐stroma ratio through the H&E staining and then predicted the prognosis in early or FIGO stage IIIC cervical cancer.^5^, ^6^ Besides, the pathological risk factors such as the human papillomavirus status and tumor immune microenvironment status were confirmed to have a significant impact on the survival rate.^7^, ^8^ With these signatures, the strategy of early or aggressive treatment may be changed for targeting patients with different prognostic effects. However, since most of these studies were based on a manually quantitative analysis, they were inevitably limited by either subjectivity or non‐repeatability.^9^

The quantitative description of the microenvironment could be used to refine the description of the pathological characterization of the tumor, and then to describe the evolution of the tumor. Along with the advantages of simplicity and rapidity, digital pathology used digital technology to digitize the pathological images to retain high‐quality image information.^10^ The whole slide image (WSI) produced by whole‐slide imaging was a great source of information in digital pathology. Compared to the traditional manual recognition of pathological images, deep learning could automatically extract more discriminative information features, with the strength of a simpler extraction process and an easily and systematically adjustable performance.^11^ Deep learning enables the pathologists to identify unique imaging markers associated with disease processes to improve early detection, determine prognosis, and select treatments that are most likely to be effective.^12^ After the WSI was input into the model, the parameters representing the intensity of cell‐to‐cell interaction could be output, and the interaction among tumor cells, stromal cells, and lymphocytes could be quantified. Since WSI can reveal the disease and related molecular progressions, the findings suggest that the deep learning techniques can be applied to WSI for accurate and objective outcome prediction of glioma patients.^13^ Moreover, deep learning framework is also an effective and labor‐saving method for WSI to provide a valuable measure for hepatocellular carcinoma risk stratification and precise patient treatment.^14^ Moreover, deep learning methods can predict microsatellite instability of patients with gastrointestinal cancer directly from WSI,^15^ and precisely identify the origin location of tumor for metastatic tumors.^16^ In addition, the combination of deep learning and WSI has been used in the prognosis prediction of lung cancer,^13^ breast cancer,^17^ and ovarian cancer,^18^ and made favorable progress. However, there were no studies on the prognosis of cervical cancer.

The purpose of our study is to discover the potential quantitative risk characteristics from the WSI, construct an effective signature in cervical cancer for prognosis prediction and patients' stratification in overall survival (OS) and disease‐free survival (DFS).

## 2 METHODS

### 2.1 Patients

This study enrolled 251 patients with cervical cancer. Two hundred and four patients were obtained from Nanfang Hospital of Southern Medical University (Guangzhou, China) from January 2009 to December 2016, the remaining 47 patients were from the other nine hospitals (Table S1). We set a descriptive analysis of the OS and DFS as valid survival endpoints. OS was defined as the time from the date of surgery to the date of death from any cause. DFS was defined as the time from the date of surgery to the date of disease recurrence or death from cervical cancer. Patients with no evidence of recurrence or death were censored at the date of the last follow up or return visit. Eligibility criteria are as follows^1^: 18 years or older^2^; FIGO stage IA1 (lymphovascular invasion, LVSI)‐IIA2 cervical cancer^3^; undergoing surgery as the initial treatment without any preoperative adjuvant treatment^4^; histological type of squamous‐cell carcinoma, adenocarcinoma or adenosquamous carcinoma^5^; undergoing abdominal Q‐M type B or C2 adical hysterectomy+ (RH+) pelvic lymphadenectomy± parabdominal aortic lymphadenectomy^6^; and all patients were followed up by more than 5 years. Exclusion criteria are^1^: Not eligible for inclusion^2^; patients had unique circumstances (e.g., pregnancy with cervical cancer, or cases complicated by another cancer)^3^; and the pathological sections are not available.

### 2.2 WSI preprocessing

WSI of all the patients was processed by HE staining; patients with only one WSI, and the tumor area was annotated by the senior pathologists (two pathologists with 3 and 5 years of clinical experience, respectively) as a region of interest (ROI). Due to the large size of WSI, we sampled the ROI area down in small patches of size 512 × 512 pixels under 40 × physical magnification considering the calculation efficiency and time cost.

The generation of WSI is susceptible to various factors, such as differences in scanning equipment, operator, staining, lighting, etc., which will cause the color difference between patients. We minimized the impact on model performance caused by the color difference of WSI through color correction (‘Staintools’ package in python) on all the sampled patches.

### 2.3 Transfer learning and autoencoder for the deep‐encode (DE) features extraction

**FIGURE 1:**
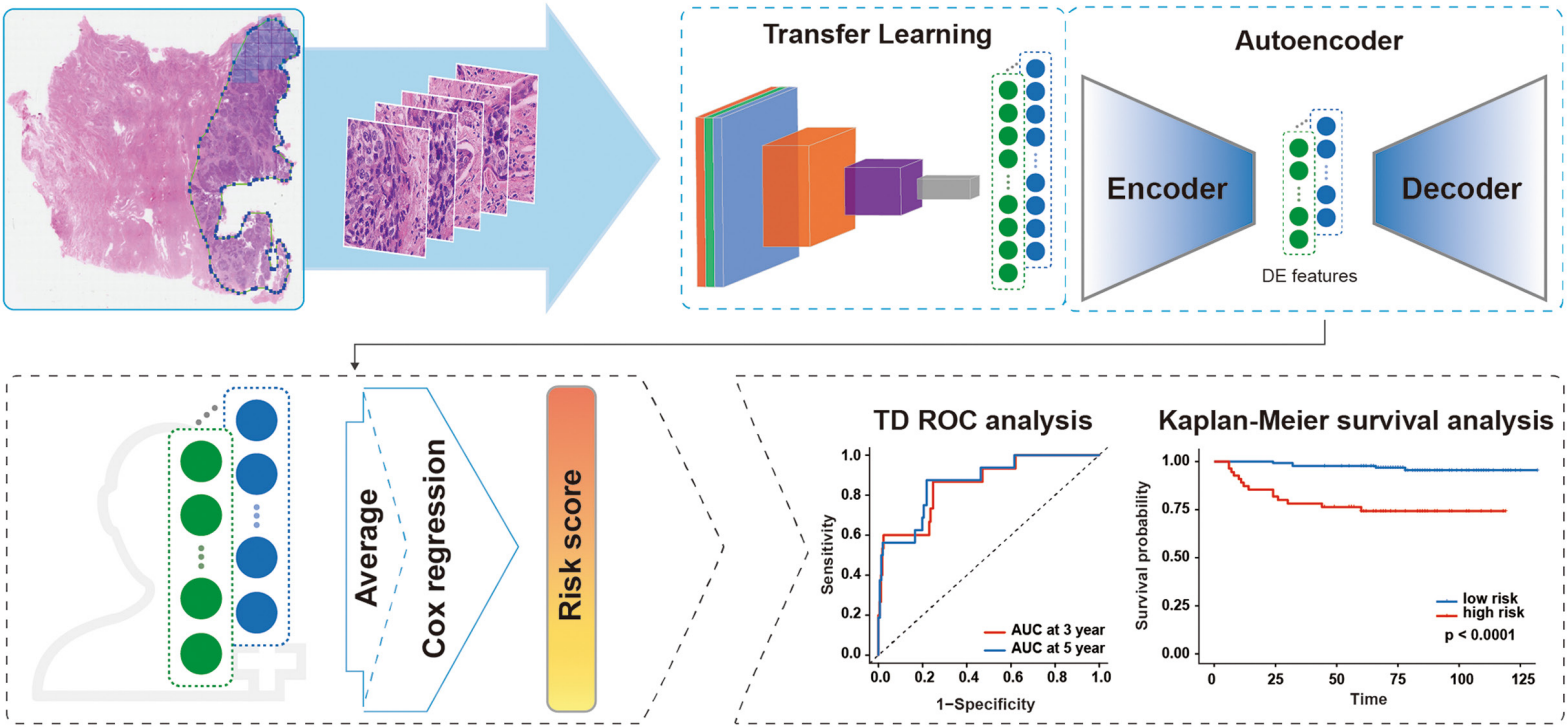
An overview of establishing and verifying RS. TD ROC analysis: Time‐dependent receiver operator characteristics analysis.

WSI has a large amount of information and often consists of many non‐artificially defined semantic features. We obtained the depth characteristics of the WSI through the method of transfer learning. For each patch, a ResNet 50 model was pre‐trained on ImageNet to extract a total of 2048‐dimensional features. (Figure 1).

An autoencoder, which consists of three hidden‐layer architectures (of 512, 128, 512 neurons) and activated by the ‘relu’ function, was constructed for the depth features dimensionality reduction and to avoid overfitting. It can embed high‐dimensional features into smaller sizes through iterative training to obtain the DE features.

A data set for auto‐encoder training and verification was constructed by randomly selecting 10% of the high‐dimensional feature data for each patient. With a batch size of 32, 150 epochs were trained, and the final result was stabilized. In the training and validation cohorts, the ‘mse’ losses reached 4.2330e‐5, 4.0814e‐5, the accuracy rates reached 93.67%, 95.50%, respectively.

### 2.4 Patient‐level DE (PDE) feature acquisition and RS building

Through the above methods, a large number of DE features were obtained from each patient. To obtain patient‐level features, we performed average processing on each dimension of DE features. All the patients were stochastically divided into training and testing cohorts at the ratio of 3:1. We randomly selected 50% of the patients on the training cohort as a feature selection subgroup, and used the elasticnet‐cox regression algorithm model to perform significant feature selection on the subgroup. Then, we repeatedly selected subgroups and filtered features to record the appearance frequency of features, and took the top 15% features with higher frequency as the salient PDE features. The PDE features were more robust than the feature filtered directly based on the whole training cohort, and could better prevent the overfitting.

To predict the survival of cervical cancer patients, Based on the PDE features of the patients, we constructed a radiomics signature on the training cohort through the multivariable Cox proportional hazards model analysis. RS was obtained by normalizing the radiomics signature. The formula is as follows:

$${\text{Score}}_{i}=\frac{{\mathrm{Sig}}_{i}}{\sqrt{\sum_{k=1}^{n}{{\mathrm{Sig}}_{k}}^{2}/n}}$$

${\text{Score}}_{i}$ is RS of the i‐th patient, ${\mathrm{Sig}}_{k}$ is the radiomics signature of the k‐th patient, and *n* is the total number of patients.

### 2.5 Validation of WSI‐predict model and RS

First, to evaluate the independent association with DFS and OS, we applied the multivariate analysis on the RS and clinical indicators. Second, to verify the performance of the image‐predict model, we calculated the Harrell's concordance index (C‐index) in both training and testing cohorts; we also constructed a baseline model based on clinical survival predictive indicators (FIGO staging, histological subtypes, vaginal margin involvement, parametrial invasion, lymph node (LN) metastasis, positive LN number, lymph vascular space invasion (LVSI), and tumor size) through the multivariable Cox proportional hazards model analysis for comparison. The Kaplan–Meier survival analysis and time‐dependent receiver operator characteristics (ROC) analysis were performed on both training and test cohorts for discovering the potential association between the RS and survival. The patients were divided into high‐ or low‐ RS groups using the X‐tile software to determine the threshold on the training cohort. Following this, the same threshold was also applied to the stratification of the testing cohort.

To further verify the performance of RS, we performed the Kaplan–Meier survival analysis on patients with clinical‐high or low‐ postoperative pathological prognostic status. Clinical‐high risk refers to patients with any high‐risk factors including the LN metastasis, parametrial invasion, positive vaginal margins, or at least two intermediate risk factors according to Sedlis standards such as LVSI, deep stromal invasion (DSI), large tumor size (large tumor size often refers to the maximum diameter of the tumor tissue, including the long diameter of tumor visible to the naked eye and the maximum diameter under the microscope). Clinical‐low risk refers to patients with no more than one medium risk factor. Meanwhile, the association of positive LN number and FIGO stage with RS were further explored by the same threshold.

### 2.6 Statistical analysis

$\chi^{2}$ test or Fisher's test was used to perform categorical variables were performed using the. In the Kaplan–Meier survival analysis, the p‐values were obtained from the log‐rank test. In addition, significant results were drawn with the *p* < 0.05. R software (version 3.6.2; https://www.r‐project.org/) was used for the statistical analysis, while the packages used for the study included survival, rms, survminer and timeROC.

## 3 RESULT

### 3.1 Patients characteristics

The characteristics of all the 251 enrolled patients are shown in Table 1. The age of all patients ranged between 29 and 75 years, the median age was 47.23 years. In the training cohort, 19 patients (10.1% of all the cohort patients) died and 37 patients (19.6% of all the cohort patients) developed metastases. In the testing cohort, s patients (11.3% of all the cohort patients) died and 12 patients (19.4% of all the cohort patients) developed metastases. The median follow‐up time for OS in training and testing cohorts were 76 months (interquartile range [IQR]: 62–96 months) and 83 months (IQR: 63.5–103.25 months); whereas, the median follow‐up time for DFS in the training and testing cohorts were 75 (IQR: 61–96 months) and 82 months (IQR: 63–103.25 months), respectively.

**TABLE 1:** Clinical characteristics of patients in training and testing cohorts

Variables	Training cohort		*p* value	Testing cohort		*p* value	Training cohort		*p* value	Testing cohort		*p* value
	Low OS‐RS	High OS‐RS		Low OS‐RS	High OS‐RS		Low DFS‐RS	High DFS‐RS		Low DFS‐RS	High DFS‐RS	
Stage			0.837			0.161			0.626			0.538
IB1	83 (72.8%)	31 (27.2%)		28 (70.0%)	12 (30.0%)		98 (86.0%)	16 (14.0%)		36 (90.0%)	4 (10.0%)	
IB2	17 (70.8%)	7 (29.2%)		1 (20.0%)	4 (80.0%)		22 (91.7%)	2 (8.3%)		5 (100.0)	0 (0.0%)	
IIA1	29 (67.4%)	14 (32.6%)		10 (71.4%)	4 (28.6%)		40 (93.0%)	3 (7.0%)		13 (92.9%)	1 (7.1%)	
IIA2	5 (62.5%)	3 (37.5%)		2 (66.7%)	1 (33.3%)		7 (87.5%)	1 (12.5%)		2 (66.7%)	1 (33.3%)	
Histological subtypes			0.514			0.415			0.140			1.000
Squamous‐cell carcinoma	125 (70.2%)	53 (29.8%)		39 (65.0%)	20 (35.0%)		157 (88.7%)	20 (11.3%)		53 (89.8%)	6 (10.2%)	
Adenocarcinoma	9 (81.8%)	2 (18.2%)		2 (100.0%)	0 (0.0%)		10 (90.9%)	1 (9.1%)		2 (100.0%)	0 (0.0%)	
Adenosquamous carcinoma	0 (0.0%)	0 (0.0%)		0 (0.0%)	1 (100.0%)		0 (0.0%)	1 (100.0%)		1 (100.0%)	0 (0.0%)	
Vaginal margin involvement			1.000			1.000			1.000			1.000
Positive	2 (66.7%)	1 (33.3%)		0 (0.0%)	0 (0.0%)		3 (100.0%)	0 (0.0%)		0 (0.0%)	0 (0.0%)	
Negative	132 (82.1%)	54 (17.9%)		41 (66.1%)	21 (33.9%)		164 (88.2%)	22 (11.8%)		56 (90.3%)	6 (9.7%)	
Parametrial invasion			1.000			1.000			1.000			1.000
Positive	2 (100.0%)	0 (0.0%)		1 (50.0%)	1 (50.0%)		2 (100.0%)	0 (0.0%)		2 (100.0%)	0 (0.0%)	
Negative	132 (70.6%)	55 (29.4%)		40 (66.7%)	20 (33.3%)		165 (88.2%)	22 (11.8%)		54 (90.0%)	6 (10.0%)	
Lymph node metastasis			0.159			0.347			1.000			0.146
Positive	22 (61.1%)	14 (38.9%)		8 (53.3%)	7 (46.7%)		32 (88.9%)	4 (11.1%)		12 (80.0%)	3 (20.0%)	
Negative	112 (73.2%)	41 (26.8%)		33 (70.2%)	14 (29.8%)		135 (88.2%)	18 (11.8%)		44 (93.6%)	3 (6.4%)	
LVSI			0.350			0.285			0.543			0.048
Positive	20 (62.5%)	12 (37.5%)		5 (50.0%)	5 (50.0%)		27 (84.4%)	5 (15.6%)		7 (70.0%)	3 (30.0%)	
Negative	114 (72.6%)	43 (27.4%)		36 (69.2%)	16 (30.8%)		140 (89.2%)	17 (10.8%)		49 (94.2%)	3 (5.8%)	
Depth of stromal invasion			0.144			0.280			0.574			1.000
≤1/2	68 (78.2%)	19 (21.8%)		11 (55.0%)	9 (45.0%)		80 (92.0%)	7 (8.0%)		18 (90.0%)	2 (10.0%)	
>1/2	57 (67.1%)	28 (32.9%)		27 (73.0%)	10 (27.0%)		75 (88.2%)	10 (11.8%)		33 (89.2%)	4 (10.8%)	
Tumor size			1.000			0.325			1.000			0.410
≤4	104 (70.7%)	43 (29.3%)		39 (68.4%)	18 (31.6%)		130 (88.4%)	17 (11.6%)		52 (91.2%)	5 (8.8%)	
>4	30 (71.4%)	12 (28.6%)		2 (40.0%)	3 (60.0%)		37 (88.1%)	5 (11.9%)		4 (80.0%)	1 (20.0%)	

*Note*: *p* values were calculated by Chi‐square test or Fisher exact test.

Abbreviations: OS, overall survival; DFS, disease‐free survival; RS, risk score; LVSI, lymph vascular space invasion.

### 3.2 Construction of RS

Five image‐predict models were built with different random repeated screening times (of 50, 100, 500, 1000, 1500 times), while C‐index was measured to evaluate the influence of the screening times on the performance of the prediction model (Table S2). Fewer times lead to the same frequency of many features and the top features could not be accurately determined. On the contrary, the implementation of more times allowed the selected features to be more stable without the problem of frequency repetition. The results showed that 1000 random screenings could get the best model performance. A total of 18 significant PDE features were screened out to construct a multivariable Cox proportional hazards model. RS values of total patients from the training and testing cohorts were calculated based on the prediction model signature.

### 3.3 Performance evaluation of RS for OS prediction

**FIGURE 2:**
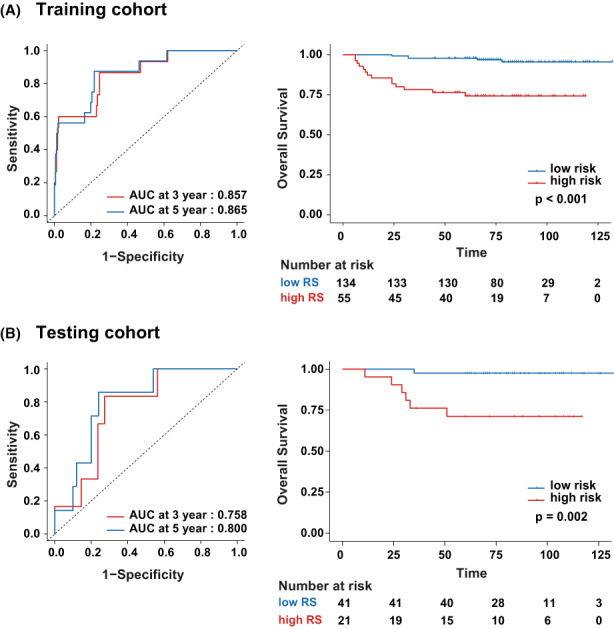
The time‐dependent ROCs and K‐M analysis of RS for overall survival in the training and testing cohort. (A) Training cohort. (B) Testing cohort. The AUCs at 3 and 5 years were calculated to assess prognosis accuracy in both cohorts. The *p*‐values calculated using the log‐rank test. AUC, area under the curve; K–M analysis, Kaplan–Meier survival analysis; OS, overall survival; ROC, receiver operator characteristic.

To evaluate the performance of RS, the C‐index of the OS prediction model was calculated, while the confidence intervals were 0.814 (0.582–0.932, 95% confidence interval [CI]) and 0.760 (0.390–0.940, 95% CI) in the training and testing cohorts, respectively. The prediction model achieved great accuracy in OS analysis. Then, the hazard ratio of RS showed significance, with 1.499 (1.297–1.732, 95% CI) in the training cohort and 1.934 (1.186–3.155, 95% CI) in the testing cohort. All the *p* values <0.001, which confirmed indicated that the RS has a significant association with the survival. Furthermore, the time‐dependent ROC analysis was applied to quantitatively evaluate the performance of RS (Figure 2). For training and testing cohorts, the 3‐year area under the curve (AUC) achieved 0.857 (0.758–0.957, 95% CI) and 0.758 (0.598–0.917, 95% CI); whereas, the 5‐year AUC achieved 0.865 (0.774–0.957, 95% CI) and 0.800 (0.659–0.942, 95% CI), respectively.

To verify the contribution of RS in the division of great‐ and poor‐prognosis groups. A threshold of 0.295 was selected through the X‐tile software. The patients were classified into a low‐risk group (RS < threshold) and a high‐risk group (RS ≥ threshold). The Kaplan–Meier survival curves of the training and test cohorts are shown in Figure 2. Patients in the high‐risk group correspond to the poor OS and vice versa (all the *p* values <0.05).

### 3.4 Performance evaluation of RS for DFS prediction

**FIGURE 3:**
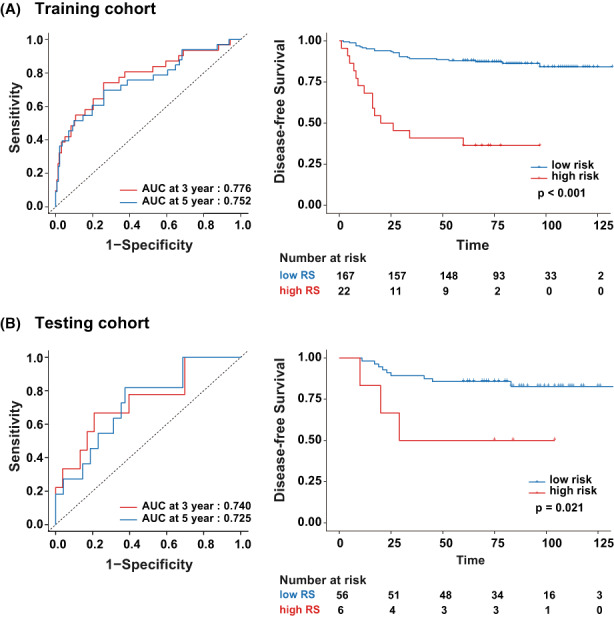
The time‐dependent ROCs and K–M analysis of RS for disease‐free survival in the training and testing cohort. (A) Training cohort. (B) Testing cohort. The AUCs at 3 and 5 years were calculated to assess prognosis accuracy in both cohorts. The *p*‐values calculated using the log‐rank test. AUC, area under the curve; DFS, disease‐free survival; K‐M analysis, Kaplan–Meier survival analysis; ROC, receiver operator characteristic.

The same analysis strategy was applied in DFS. All the analyses had an advantageous outcome. First, the C‐index of the DFS‐prediction model was evaluated, with 0.738 (0.566–0.858, 95% CI), 0.709 (0.412–0.894, 95% CI) in the training and testing cohorts, respectively. Furthermore, a potential association with prognosis was demonstrated, while the hazard ratio of RS achieved 2.031 (1.641–2.515, 95% CI) in the training cohort and 3.367 (1.748–6.487, 95% CI) in the testing cohort. We then performed the time‐dependent ROC in both cohorts (Figure 3), with the 3‐year AUC of 0.776 (0.677–0.876, 95% CI) and 0.740 (0.557–0.923, 95% CI), and the 5‐year AUC of 0.752 (0.649–0.854, 95% CI) and 0.725 (0.568–0.883, 95% CI) in the training and testing cohorts, respectively.

Moreover, similar to the OS analysis, with the threshold of 1.310 calculated by the X‐tile software, the patients were divided into the low‐ or high‐risk groups. The Kaplan–Meier survival curves analysis was performed and shown in Figure 3.

### 3.5 Performance of RS according to clinical subtypes

**FIGURE 4:**
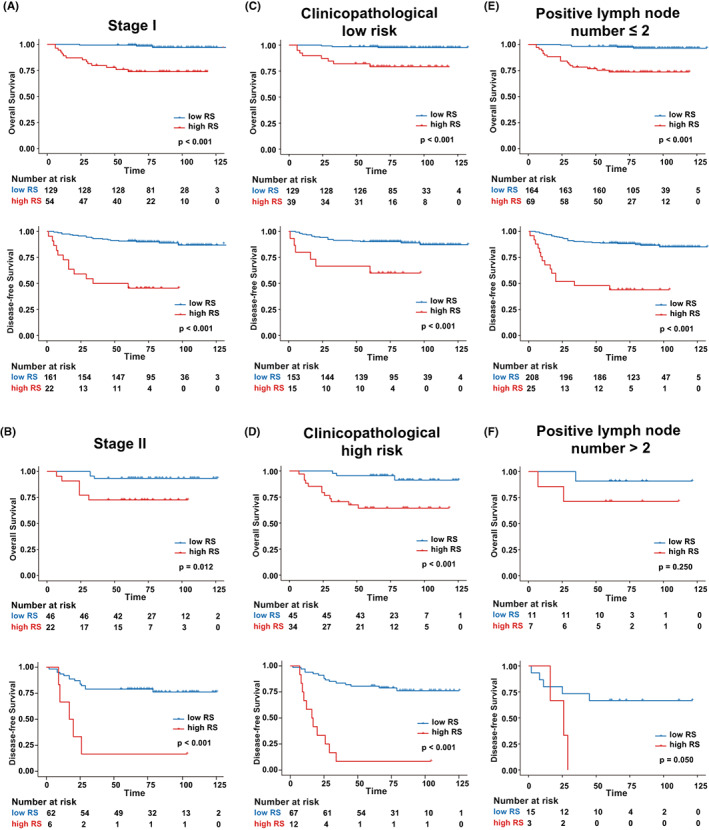
The results for the performance of RS according to the clinical subtypes. Kaplan–Meier survival analysis of overall survival and disease‐free survival according to RS classifier in subgroup patients stratified by FIGO stage, clinicopathological high‐ and low‐risk factors, and positive lymph node number.

We then performed the Kaplan–Meier survival analysis by the same threshold of RS according to different FIGO stages, clinicopathological risk status, and positive LN number (Figure 4). The results illustrated that the significant DFS and OS difference between the high‐ and low‐RS groups persisted among different FIGO stages (Figure 4A, Figure 4B). Moreover, RS was able to further distinguish significant survival differences between high‐ or low‐RS regardless of the clinicopathological high‐risk status (Figure 4C, Figure 4D). Although RS failed to achieve good performance when patients with positive LN numbers larger than 2, it may be largely due to the too‐small dataset with only 16 patients enrolled.

### 3.6 Performance comparison evaluation with the clinical characteristics

We verified that the RS was an independent prognostic factor associated with the survival rate (Table 2, Table 3). Furthermore, based on the manual clinical characteristics, the baseline prediction model was constructed, while time‐dependent ROC analysis was performed to assess the performance of the prognosis prediction. The 3‐year and 5‐year AUC values for both OS and DFS analysis were calculated (Table 4). The overfitting appeared for applying DFS analysis on 5‐year AUC. Furthermore, RS showed the advantage compared to the baseline characteristics on predicting OS and DFS.

**TABLE 2:** Multivariable analysis of the risk score and clinical indicators with disease‐free survival and overall survival in the training cohort

	Overall survival		Disease‐free survival	
	HR (95% CI)	*p* value	HR (95% CI)	*p* value
Risk score	1.667 (1.349–2.059)	<0.001	2.205 (1.716–2.834)	<0.001
Age	0.994 (0.935–1.055)	0.836	1.005 (0.964–1.047)	0.827
FIGO stage		0.657		0.011
IB1	Reference		Reference	
IB2	0.698 (0.127–3.851)	0.680	3.078 (0.781–12.134)	0.108
IIA1	0.770 (0.192–3.080)	0.711	2.832 (1.242–6.457)	0.013
IIA2	3.244 (0.513–20.521)	0.211	2.691 (0.451–16.070)	0.278
Histological subtypes	4.368 (1.201–15.888)	0.025	2.350 (0.694–7.957)	0.170
Lymph node metastasis	0.282 (0.048–1.646)	0.160	1.176 (0.296–4.676)	0.818
Positive lymph node number	0.926 (0.413–2.080)	0.853	1.320 (0.755–2.309)	0.330
LVSI	2.195 (0.429–11.217)	0.345	0.776 (0.322–1.868)	0.571
Tumor size	0.786 (0.187–3.313)	0.743	0.488 (0.144–1.649)	0.248

Abbreviations: CI, confidence interval; HR, hazard ratio; LVSI, lymph vascular space invasion.

*Note*: *p* values were calculated by likelihood ratio test.

**TABLE 3:** Univariate analysis of the risk score and clinical indicators with disease‐free survival and overall survival in the training cohort

	Overall survival		Disease‐free survival	
	HR (95% CI)	*p* value	HR (95% CI)	*p* value
Risk score	1.499 (1.297–1.732)	<0.001	2.031 (1.641–2.515)	<0.001
Age	0.997 (0.945–1.051)	0.897	1.024 (0.987–1.062)	0.210
FIGO stage		0.863		0.036
IB1	Reference		Reference	
IB2	0.768 (0.172–3.431)	0.730	1.448 (0.534–3.928)	0.467
IIA1	0.654 (0.185–2.319)	0.511	2.201 (1.069–4.534)	0.032
IIA2	2.791 (0.624–12.485)	0.179	1.929 (0.445–8.351)	0.380
Histological subtypes	3.455 (1.006–11.870)	0.049	1.605 (0.492–5.230)	0.433
Lymph node metastasis	0.486 (0.185–1.278)	0.143	0.590 (0.286–1.221)	0.155
Positive lymph node number	1.208 (0.821–1.778)	0.338	1.235 (0.926–1.649)	0.151
LVSI	0.721 (0.239–2.176)	0.562	0.494 (0.239–1.022)	0.057
Tumor size	0.923 (0.306–2.780)	0.886	0.796 (0.349–1.813)	0.587

*Note*: *p* values were calculated by likelihood ratio test.

Abbreviations: CI, confidence interval; HR, hazard ratio; LVSI, lymph vascular space invasion.

**TABLE 4:** AUC for baseline score and risk score at 3, 5 years in the training and testing cohorts

	Overall survival		Disease‐free survival	
	3‐year AUC (95% CI)	5‐year AUC (95% CI)	3‐year AUC (95% CI)	5‐year AUC (95% CI)
Training cohort				
Baseline score	0.684 (0.561–0.807)	0.648 (0.512–0.785)	0.673 (0.568–0.778)	0.636 (0.524–0.747)
Risk score	**0.857 (0.758–0.957)**	**0.865 (0.774–0.957)**	**0.776 (0.677–0.876)**	**0.752 (0.649–0.854)**
Testing cohort				
Baseline score	0.530 (0.263–0.798)	0.604 (0.346–0.861)	0.713 (0.463–0.963)	**0.759 (0.548–0.971)**
Risk score	**0.758 (0.598–0.917)**	**0.800 (0.659–0.942)**	**0.740 (0.557–0.923)**	0.725 (0.568–0.883)

Bold value indicates higher value of AUC bewteen baseline score and risk score. Abbreviations: AUC, area under the curve; CI, confidence interval.

## 4 DISCUSSION

This study proposes an RS value, which is a factor independent of the main clinical characteristics, to implement survival analysis in cervical cancer patients. With stratified the patients into high‐ and low‐risk group depends on the RS value, the significant differences in DFS and OS will illustrate. In the individualized DFS and OS estimation, RS performed better than the important clinical prognostic indicators.

In our study, RS was able to predict the survival better than the clinicopathological characteristics, based on the larger C‐index values in both two cohorts. It could due to the two main reasons. Firstly, clinical factors only reflect the manual tumor information, which depends on the prior knowledge, while a large number of semantic structural tumor features are ignored. Secondly, the high‐dimensional deep learning imaging features, which were extracted from the WSI, could provide additional details regarding the tumor microenvironment properties that allowed for more comprehensive characterization of the prognostic information.^19^

Currently, individual prognostic stratification after surgery mostly relies on the clinicopathological factors. Patients who are routinely administered with adjuvant chemoradiation to improve survival, and those with postoperative pathological high‐risk factors (positive surgical margin, parametrial involvement, and LN metastasis^20^, ^21^) or two or more intermediate‐risk factors (LVSI, large tumor size, and deep stromal invasion^22^, ^23^) are considered to be at a greater risk of recurrence.^24^ However, there is still a lack of further risk stratification among these clinicopathological low‐ and high‐risk groups. Thanks to RS based on the PDE features, we successfully stratified the patients further into two risk categories with significant survival differences after clinicopathological stratification (Figure 4B). Cervical cancer patients with high‐RS values tend to progress or die early after surgery. Therefore, when the pathological factors are not enough to identify the survival estimation, RS can be an individual factor to assist the doctors in decision making. Furthermore, compared to the routine manual pathological judgment, our method had advantages in automation and objectivity, could save human resources and reduce subjective errors.

Besides, genes are widely used as biomarkers to predict the prognosis of cervical cancer patients. Previous studies have proved that some genes had a significant association with cervical cancer prognosis. The expression of some key genes including lncRNA ZFAS1,^25^ lncRNA AFAP1‐AS1,^26^ and a few MicroRNA^27^ was also confirmed to be potentially connected with the prognosis of cervical cancer. Yang et al^28^ constructed a signature based on the immune‐related genes to predict survival. Wang et al^29^ constructed a cervical cancer prognostic model based on 10 immune‐related genes (APOD, TFRC, GRN, CSK, HDAC1, NFATC4, BMP6, IL17RD, IL3RA, and LEPR), while AUC of 0.738 in the TCGA public database. Tian et al^30^ identified that the cervical cancer patients with five notable nonsynonymous mutant genes (PIK3CA, BRAF, GNA11, FBXW7, and CDH1) metastatic relapse significantly mutated mutations had significantly poor DFS and OS. However, gene expression detection has some limitations in terms of the clinical source, clinical universality, and patient acceptable cost and other factors, while it leads to additional consumption of clinical resources, the cost of medical treatment proves to be high for patients. With the development of whole‐slide imaging, the entire slide analysis becomes convenient and efficient in contrast.^12^

Additionally, some studies constructed the prognosis prediction model based on the combination of the medical image and machine learning algorithm; furthermore, they obtained significant clinical application effects. To provide the features for building random survival forest models to predict the prognosis of node‐positive cervical cancer, the primary tumor and T1‐weighted images, as well as T2‐weighted images, were saved as 3‐dimensional ROI.^31^ The combined model based on the intratumoral zone of T1‐weighted images, intratumoral zone of T2‐weighted images, and peritumoral zone of T2‐weighted images was able to predict the response to neoadjuvant chemotherapy in locally advanced cervical cancer.^32^ Furthermore, Wu et al^33^ established an end‐to‐end deep learning model to predict lymph node metastasis using sagittal contrast‐enhanced T1‐weighted imaging, axial T2‐weighted imaging, and axial diffusion‐weighted imaging. While magnetic resonance imaging achieved an advantageous result, Li et al^34^ used the radiomics features extracted from the PET‐CT to construct a model to predict E‐cadherin expression, allowing for evaluation of the patient's prognosis. Our pathological RS has been verified to be an independent prognostic indicator, which can be combined with these imaging indicators to construct a more favorable prediction model. Because the combined analysis method from macro to micro is helpful in refining the description of tumor heterogeneity.

Compared with many other imaging modalities, the complexity of WSI is higher because of its large size, color information, no apparent anatomical orientation as in radiology, availability of information at multiple scales, and multiple z‐stack levels.^12^ This makes it difficult for the human reader to extract the large source of visual information. With the development of the deep learning algorithm, the use of deep neural networks to extract WSI features has been applied in various cancer studies, while those features reflect abstract, high‐dimensional tumor image information. By combining both radiological and pathological information of the local lesions, the deep model can potentially predict pathological response to neoadjuvant chemoradiotherapy in rectal cancer.^35^ Moreover, WSI has been explored to distinguish susceptive responders,^36^ classify molecular subtype,^37^ or predict cancer survival outcomes.^9^ Those progressive studies illustrated the strong potential of WSI in tumor diagnosis and treatment.

In addition, there are some limitations in our study. First, we applied the method of features extraction, an end‐to‐end training model is hoped to be established in the future. Second, the RS and manual features could be combined for a more comprehensive signature, thus, improving the interpretability. Furthermore, owing to the inclusion and exclusion criteria, some hospitals collect uneven proportions of data, and we prospect to collect larger and more balanced training samples to support validation on more independent data sets.

In conclusion, RS can effectively predict the prognosis of cervical cancer patients, and precisely and independently divide them into different groups based on different prognosis effects. Moreover, more accurate and potential signature may become a tool to guide individual care.

## AUTHORS CONTRIBUTIONS

Chi Chen, Yuye Cao, Zhenyu Liu, Xin Tian involved in study conception and design. Chunlin Chen, Weili Li, Ping Liu, Wuliang Wang, Shan Kang, Shaoguang Wang carried out data collection. Jie Tian, Jingying Jiang, Chi Chen, Caixia Sun, Han Gao involved in analysis and interpretation of results. Chi Chen, Yuye Cao, Weili Li carried out draft manuscript preparation. All authors reviewed the results and approved the final version of the manuscript.

## CONFLICT OF INTEREST

The authors declare no conflict of interest. The funders had no role in the design of the study; in the collection, analyses, or interpretation of data; in the writing of the manuscript, or in the decision to publish the results.

## ETHICAL APPROVAL STATEMENT

This retrospective multicenter study was conducted in accordance with the Declaration of Helsinki. The study's design was approved by each institutional review board and waived the informed consent requirement.

## Supporting information


Table S1
Table S2

## Data Availability

Research data are not shared.
