# Supplementary material for: The pathological risk score: A new deep learning‐based signature for predicting survival in cervical cancer
Source: Cancer Med. 2022 Jun 28;12(2):1051–63. doi: 10.1002/cam4.4953 (PMC9883425; doi:10.1002/cam4.4953)
Supplement: Supplementary file 1 — Table S1 Table S2 [file CAM4-12-1051-s001.docx]

**Supplementary Tables**

**Table S1.** WSI source for the patients

| Hospital | Numbers of Patients | Numbers of WSI |
| --- | --- | --- |
| Nanfang Hospital of Southern Medical University | 204 | 204 |
| The First Affiliated Hospital of USTC | 5 | 5 |
| The Fourth Hospital of Hebei Medical University | 8 | 8 |
| Xinxiang Medical University | 4 | 4 |
| Qingdao Municipal Hospital | 1 | 1 |
| The Affiliated Hospital of Qingdao University | 5 | 5 |
| Second Hospital of Shanxi Medical University | 2 | 2 |
| Yantai Yuhuangding Hospital | 6 | 7 |
| The First Affiliated Hospital of Zhengzhou University | 1 | 1 |
| The Second Affiliated Hospital of Zhengzhou University | 15 | 15 |
| Total | 251 | 251 |

Abbreviations: WSI, whole slide image

**Table S2.** Prediction performance comparison between the models with different numbers of random repeated screening times.

|  | Times | | | | |
| --- | --- | --- | --- | --- | --- |
|  | 50 | 100 | 500 | **1000** | 1500 |
| C-index for OS |  |  |  |  |  |
| Training cohort | 0.831 | 0.817 | **0.819** | 0.814 | 0.814 |
| Testing cohort | 0.642 | 0.696 | 0.743 | **0.760** | 0.760 |
| C-index for DFS |  |  |  |  |  |
| Training cohort | 0.734 | 0.750 | 0.738 | **0.738** | 0.738 |
| Testing cohort | 0.730 | 0.716 | 0.709 | **0.709** | 0.709 |

Abbreviations: OS, overall survival; DFS, disease-free survival; C-index, Harrell's concordance index
